# Preservation of thalamic neuronal function may be a prerequisite for pain perception in diabetic neuropathy: A magnetic resonance spectroscopy study

**DOI:** 10.3389/fpain.2022.1086887

**Published:** 2023-01-06

**Authors:** Rajiv Gandhi, Dinesh Selvarajah, Gordon Sloan, Marni Greig, Iain D. Wilkinson, Pamela J. Shaw, Paul Griffiths, Solomon Tesfaye

**Affiliations:** ^1^ Diabetes Research Unit, Sheffield Teaching Hospitals NHS Foundation Trust, Sheffield, United Kingdom; ^2^ Department of Oncology and Human Metabolism, University of Sheffield, Sheffield, United Kingdom; ^3^ Academic Unit of Radiology, University of Sheffield, Sheffield, United Kingdom; ^4^ Sheffield Institute for Translational Neuroscience, University of Sheffield, Sheffield, United Kingdom

**Keywords:** diabetic neuropathy, painful diabetic neuropathy, neuropathic pain, magnetic resonace spectroscopy, thalamus, peripheral neuropathy

## Abstract

**Introduction:**

In this study, we used proton Magnetic Resonance Spectroscopy (1H-MRS) to determine the neuronal function in the thalamus and primary somatosensory (S1) cortex in different subgroups of DPN, including subclinical- and painful-DPN.

**Method:**

One-hundred and ten people with type 1 diabetes [20 without DPN (no-DPN); 30 with subclinical-DPN; 30 with painful-DPN; and 30 with painless-DPN] and 20 healthy volunteers, all of whom were right-handed men, were recruited and underwent detailed clinical and neurophysiological assessments. Participants underwent Magnetic Resonance Imaging at 1.5 Tesla with two 1H-MRS spectra obtained from 8 ml cubic volume voxels: one placed within left thalamus to encompass the ventro-posterior lateral sub-nucleus and another within the S1 cortex.

**Results:**

In the thalamus, participants with painless-DPN had a significantly lower NAA:Cr ratio [1.55 + 0.22 (mean ± SD)] compared to all other groups [HV (1.80 ± 0.23), no-DPN (1.85 ± 0.20), sub-clinical DPN (1.79 ± 0.23), painful-DPN (1.75 ± 0.19), ANOVA *p* < 0.001]. There were no significant group differences in S1 cortical neurometabolites.

**Conclusion:**

In this largest cerebral MRS study in DPN, thalamic neuronal dysfunction was found in advanced painless-DPN with preservation of function in subclinical- and painful-DPN. Furthermore, there was a preservation of neuronal function within the S1 cortex in all subgroups of DPN. Therefore, there may be a proximo-distal gradient to central nervous system alterations in painless-DPN, with thalamic neuronal dysfunction occurring only in established DPN. Moreover, these results further highlight the manifestation of cerebral alterations between painful- and painless-DPN whereby preservation of thalamic function may be a prerequisite for neuropathic pain in DPN.

## Introduction

Diabetic peripheral neuropathy (DPN) is the most common chronic complication of diabetes mellitus and is the primary risk factor for foot ulceration, which can often precipitate lower limb amputation (1). Up to half of people with DPN suffer with chronic neuropathic pain (painful-DPN), which often leads to psychological morbidity, unemployment and a reduced quality of life (2, 3). Unfortunately, our understanding of the pathophysiology of painful-DPN remains inadequate. Consequently, current treatments for the condition target neuropathic symptoms rather than the underlying mechanistic changes (2). These pharmacotherapeutic agents are unsatisfactory, resulting in only modest pain relief which is often offset by intolerable side effects (4). A further understanding of the pathophysiology of neuropathic pain in diabetes is therefore essential for us to identify new targets for therapeutic interventions.

There is increasing evidence that central nervous system is involved in DPN (5). A number of advanced magnetic resonance imaging (MRI) techniques have been used to offer valuable insights into the cerebral alterations present in DPN in several regions of the brain responsible for somatosensory function (6–11). The thalamus and the primary somatosensory (S1) cortex are two such brain regions, both of which are critical for the processing of noxious pain signals. The ventero-posterior nucleus of the thalamus receives and modulates nociceptive impulses from ascending sensory pathways before transmitting signals to other areas of the brain, such as the S1 cortex (12). The S1 cortex is then integral for the localization and perception of pain.

The investigative technique in the study was proton magnetic resonance spectroscopy (1H-MRS). 1H-MRS is an imaging technique used to interrogate the neurochemistry of a particular region of interest, known as a voxel (13, 14). Our group have previously used this technique to demonstrate that levels of thalamic N-acetyl aspartate (NAA), a marker of neuronal health, viability and number (13), were reduced in patients with type 1 diabetes and painless-DPN compared to non-neuropathic patients with diabetes and healthy volunteers (15). These results are suggestive of neuronal dysfunction at the thalamus in painless-DPN. However, the neuronal function in other phenotypes of DPN and different brain regions remains understudied. We conducted a study to determine the neuronal function in the thalamus and S1 cortex in different subgroups of DPN, including subclinical- and painful-DPN.

## Method

### Participants

The study included 130 right-handed men [110 with type 1 diabetes and 20 healthy volunteers (HV)]. Inclusion criteria were: type 1 diabetes for >5 years, male, right-handedness, age between 18 and 65 years and willingness to discontinue neuropathic pain medications before MRI scan. Exclusion criteria were: clinical evidence of disease in the central nervous system (e.g., cerebrovascular disease), non-diabetic neuropathies, history of alcohol consumption of >20 units/week (1 units is equivalent to 1 glass of wine or 1 measure of spirits), diabetic neuropathies other than DPN (e.g., mononeuropathies, proximal motor neuropathies), psychiatric conditions, claustrophobia or other factors that preclude MRI. Written informed consent was obtained before participation in the study, which had prior approval by the South Sheffield Regional ethics committee.

### Clinical assessment

All participants underwent detailed clinical and neurophysiological assessments. The outcome of a detailed upper- and lower-limb neurological examination was graded using the Neuropathy Impairment Score clinical scoring system (16). The following neurophysiological assessments were performed: vibration detection thresholds acquired from the dorsal aspect of the right foot using computer-assisted sensory evaluation (CASE IV; W.R. Electronics, Stillwater, MN, USA) using standard techniques (17); cardiac autonomic function tests performed with a computer-assisted technique according to O’Brien's protocol (18); and nerve conduction studies performed at a stable skin temperature of 31°C and a room temperature of 24°C, using an electrophysiological system (Medelec; Synergy Oxford Instruments, Oxford, UK). An overall composite score [NIS(LL) + 7] derived from transformed percentile points of abnormalities in nerve conduction studies, vibration detection thresholds, and heart rate variability with deep breathing was calculated in accordance with criteria proposed by Dyck et al. (19).

On the basis of clinical and neurophysiological assessments, participants with diabetes were divided into four groups according to the Toronto consensus recommendations (20):

(i) No-DPN: consisting of patients with diabetes without features of subclinical, painless or painful-DPN
(ii) Subclinical-DPN: consisting of patients with diabetes without symptoms or signs of DPN but two or more abnormalities on neurophysiological assessments
(iii) Painless-DPN: consisting of pain-free patients with diabetes with clinical signs/symptoms of DPN and at least two abnormalities on neurophysiological assessment
(iv) Painful-DPN: consisting of patients with painful neuropathic symptoms and diabetes with clinical signs/symptoms of DPN and at least two abnormalities on neurophysiological assessment.

### Magnetic resonance spectroscopy

In order to avoid pharmacotherapeutic agents causing alterations in brain neurochemistry, anti-depressants and anticonvulsants were discontinued for 2 weeks prior to scanning. Paracetamol, aspirin, non-steroidal anti-inflammatory medications and weak opioids were allowed for breakthrough analgesia, if required.

**Figure 1:**
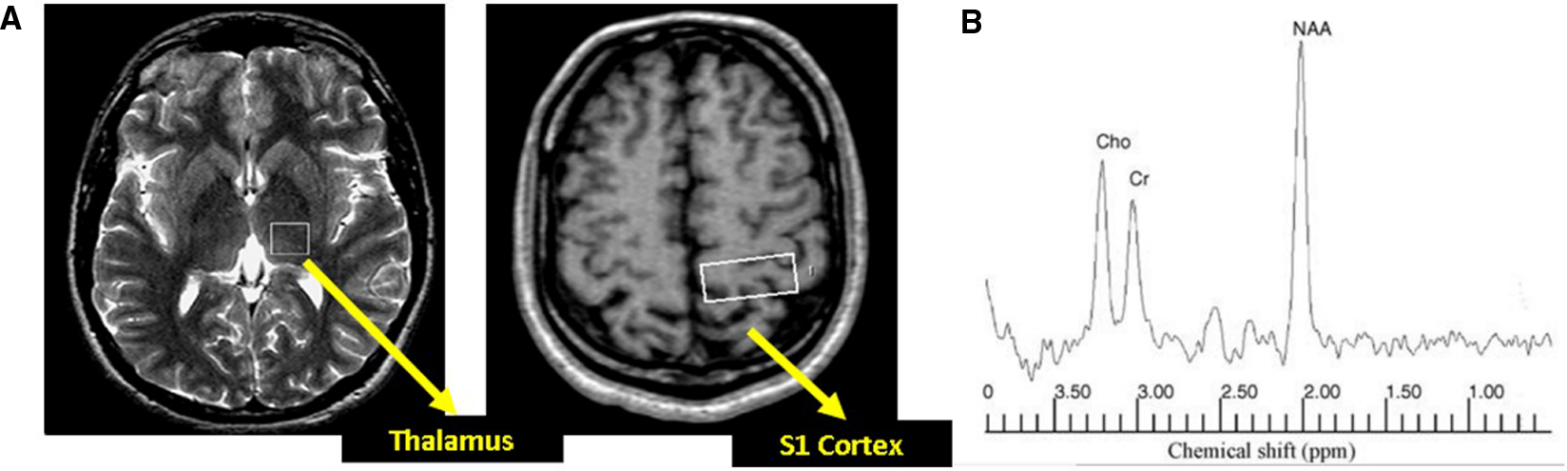
(**A**) Axial section of the brain with voxel positioning in the thalamus and primary somatosenxory (S1) cortex. (**B**) Example of spectra obtained using a point-resolved acquisition technique. NAA, N-acetyl aspartate; Cho, choline; Cr, creatine.

Participants underwent 1H-MRS at 1.5 T (Eclipse; Philips Medical Systems, Cleveland, OH, USA). Single-voxel spectra were obtained from an 8 ml cubic volume in two regions of interest (ROI) placed within the left thalalmus to encompass the ventro-posterior lateral sub-nucleus and a second ROI within the post-central gyrus (S1 cortex) (Figure 1). Spectra were acquired at each ROI as previously described (15) [echo time (TE) = 135 ms, resonance time = 1,600 ms] using a point-resolved technique.

MR spectra were independently and anonymously analysed by an experienced MR physicist (IDW). All post acquisition processing was performed using fully integrated proprietary software from the manufacturer of the MR system. By convention, results are expressed as ratios under the three prominent resonances assigned to Cho (3.22 ppm), Cr (3.02 ppm) and NAA (2.02 ppm) ie. NAA:Cho; NAA:Cr and Cho:Cr ratios.

### Statistical analysis

Statistical analysis was performed using the statistical package SPSS 14 (SPSS Chicago, IL, USA). Baseline characteristics and study endpoints were described as means and standard deviations for normally distributed variables and as medians and range for variables with a skewed distribution.

The appropriate tests for normality were conducted to guide subsequent analysis. Subgroup 1H-MRS metabolite endpoints were compared using parametric tests (ANOVA).

## Results

The demographic, clinical and neurophysiological data of studied patients are presented in Table 1. The participants in the no-DPN and subclinical-DPN groups were significantly younger than both clinical DPN groups. Additionally, the subclinical-DPN group had a shorter duration of diabetes compared with those with painless-DPN (ANOVA, *p* < 0.01), but not the other diabetes groups. HbA1c was higher in the painless-DPN group compared with no-DPN (ANOVA, *p* < 0.05) and systolic blood pressure was greater in the painless-DPN group compared with no-DPN and subclinical-DPN groups. There were no statistically significant differences in the age, BMI, duration of DM, HbA1c, blood pressure or neuropathy severity between the painful- and painless-DPN groups.

**Table 1:** Demographic, clinical and neurophysiological characteristics of study participants.

	HV (*n* = 20)	No-DPN (*n* = 20)	Subclinical-DPN (*n* = 30)	Painful-DPN (*n* = 30)	Painless-DPN (*n* = 30)	*p*-value (ANOVA)
Age (years)	53 ± 15.2	46 ± 9.4	43 ± 7.1	55 ± 13.1	55 ± 10.4	*p* < 0.001
BMI (kg/m^2^)	26.6 ± 4.1	27.0 ± 4.2	25.2 ± 3.9	28.7 ± 4.4	28.3 ± 4.4	NS
Duration of diabetes (years)	-	23 ± 12.3	18 ± 7.6	24 ± 9.5	27 ± 10.9	*p* < 0.001
HbA1c (%)	-	7.7 ± 1.2	8.3 ± 1.0	8.5 ± 1.5	8.8 ± 1.6	*p* < 0.05
SBP (mmHg)	139 ± 14	134 ± 12	134 ± 10	143 ± 19	152 ± 18	*p* < 0.001
NIS (LL) + 7 Score)	0.4 ± 0.7	1.4 ± 1.2	4.3 ± 0.9	21.1 ± 10.8	17.2 ± 7.0	*p* < 0.001

BMI, body mass index; SBP, systolic blood pressure; NIS(LL) + 7, Neuropathy Impairment Score of the Lower Limbs plus 7 neurophysiological tests.

### Magnetic resonance spectroscopy of the thalamus and primary somatosensory cortex

**Figure 2:**
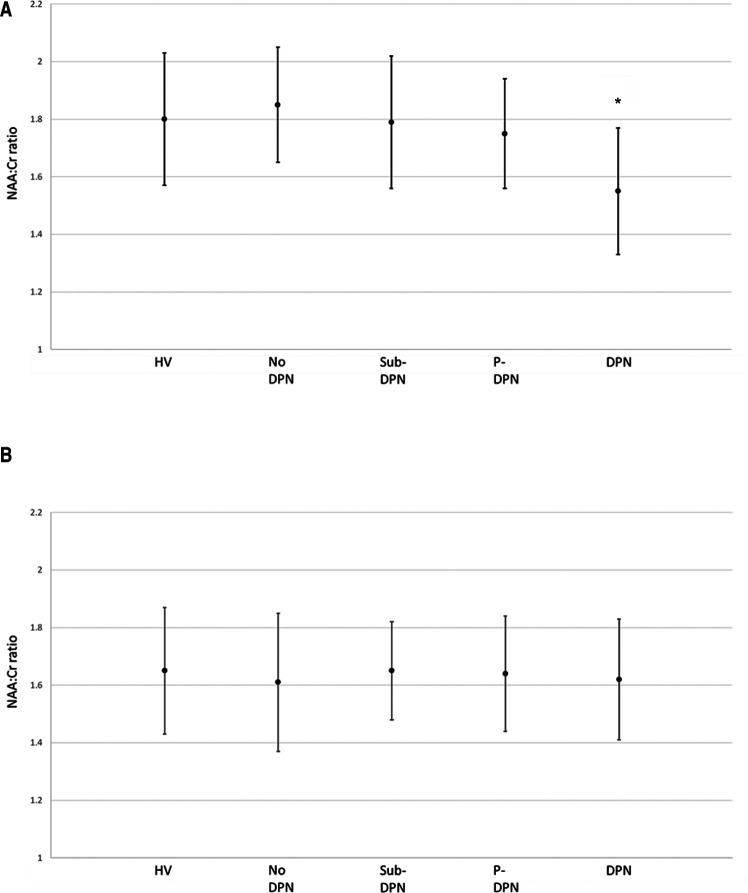
NAA:Cr ratio and 95% CI at the (**A**) thalamus and (**B**) primary somatosensory cortex. *p = <0.001. DPN, painless-DPN; HV, healthy volunteer; No DPN, diabetes without DPN; P-DPN, painful-DPN; Sub-DPN, subclinical-DPN.

Spectroscopy results at both ROI are displayed in Table 2. In the thalamic ROI, participants with painless DPN had a significantly lower NAA:Cr ratio [1.55 + 0.22 (mean ± SD)] compared to all other groups [HV (1.80 ± 0.23), no DPN (1.85 ± 0.20), sub-clinical DPN (1.79 ± 0.23), painful-DPN (1.75 ± 0.19), ANOVA *p* < 0.001] (Figure 2A). However, there were no significant differences in NAA:Cho or Cho:Cr ratios in the thalamus. At the S1 cortex ROI, there were no significant group differences in NAA:Cr, NAA:Cho or Cho:Cr (Figure 2B).

**Table 2:** Magnetic resonance spectroscopy measurements in the thalamus and primary somatosensory cortex.

	HV	No DPN	Subclinical DPN	Painful DPN	Painless DPN	*p*-value (ANOVA)
Thalamus						
NAA:Cr	1.80 ± 0.23	1.85 ± 0.20	1.79 ± 0.23	1.75 ± 0.19	1.55 ± 0.22	<0.001
NAA:Cho	1.92 ± 0.31	1.91 ± 0.28	1.87 ± 0.25	1.74 ± 0.25	1.75 ± 0.26	ns
Cho:Cr	0.95 ± 0.12	0.98 ± 0.13	0.95 ± 0.17	1.00 ± 0.17	0.92 ± 0.16	ns
Primary somatosensory cortex						
NAA:Cr	1.65 ± 0.22	1.61 ± 0.24	1.65 ± 0.17	1.64 ± 0.20	1.62 ± 0.21	ns
NAA:Cho	2.19 ± 0.26	2.22 ± 0.48	2.24 ± 0.36	2.14 ± 0.30	2.14 ± 0.31	ns
Cho:Cr	0.76 ± 0.10	0.75 ± 0.13	0.75 ± 0.11	0.78 ± 0.13	0.77 ± 0.12	ns

NAA, N-acetyl aspartate; Cr, creatine and phosphocreatine; Cho, choline.

## Discussion

The main finding of this large 1H-MRS study is that patients with painless-DPN have a reduced NAA:Cr ratio, whilst those with subclinical- and painful-DPN have a ratio comparable to people with diabetes without neuropathy and healthy volunteers. Moreover, we found that S1 cortex 1H-MRS neurometabolites were unaffected in all diabetes subgroups and were not different to healthy volunteers.

NAA is one of the most abundant molecules within the central nervous system and is synthesized within the neuronal mitochondria (13). It is a precursor to the neuropeptide N-Acetyl-aspartyl-glutamate and is involved in neuronal osmoregulation, myelin lipid synthesis and assisting nitrogen removal from the brain. NAA is well recognized as an 1H-MRS marker of neuronal viability and function. Reduced cerebral NAA levels have been demonstrated in a number of neurological disorders associated with neuronal dysfunction, including ischaemic stroke (21), neurodegenerative dementias (22), multiple sclerosis (23) and psychiatric disorders (24). Moreover, reduced NAA levels have also been demonstrated in a number of brain regions in patients with type 1 and type 2 diabetes mellitus and associated diabetic complications, including DPN (11, 15, 25–27). The findings of this study are indicative of thalamic neuronal dysfunction in patients with painless-DPN, but not other phenotypes of DPN. This finding is consistent with our previous research (15), but is now confirmed in a considerably larger cohort. Other groups have also recently identified thalamic gray matter volume loss (28) and/or reduced cerebral NAA:Cr (10, 27) in DPN. However, these studies have not included a diabetes control group. Our study included a no-DPN group, therefore confirming that the reduction in NAA:Cr is linked to the neuropathic process and is not just a “diabetes-effect” upon the brain.

The underlying mechanism of thalamic neuronal dysfunction in painless-DPN remains unclear. We have previously hypothesised that a loss of afferent input in the DPN may cause dying back of central nervous system neurons. Our group demonstrated sluggish blood flow to the thalamus in painless- compared with painful-DPN, indicating a reduction in thalamic neuronal activity (6). Moreover, several groups have shown a reduction in thalamic gray matter volume in DPN/painless-DPN (10, 28, 29). A recent study for example used voxel based morphometry to measure brain volumetry and found a reduction in gray matter volume in the thalamus in patients with DPN compared to healthy volunteers (10). Notably they also found that intra-thalamic NAA:Cr correlated with thalamic volume, thereby suggesting a corresponding loss of neuronal function and volume which may indicate thalamic neuronal loss. The limitation of all the published brain imaging studies in DPN is their cross-sectional study design. Future studies need to have a longitudinal design to explore the mechanisms underlying these thalamic alterations in DPN.

This study is one of the few to examine the central nervous system alterations in participants with subclinical-DPN. Previously, we have demonstrated a reduction in the cross-sectional area of the spinal cord in patients with subclinical-DPN compared to healthy volunteers and no-DPN but to a lesser degree than those with more advanced clinical-DPN (30). However, in this study we found that thalamic metabolites were unaltered in subclinical-DPN compared with healthy volunteers and no-DPN. Therefore, thalamic dysfunction may be a later occurrence than spinal cord area loss in painless-DPN. This suggests that there may be a proximo-distal gradient to central nervous system alterations, analogous to the peripheral nervous system, although the latter changes are predominant. The finding of unaltered S1 cortical 1H-MRS metabolite ratios in all groups is further supportive of the fact that central nervous involvement in DPN may occur in a proximo-distal gradient. Other studies have demonstrated a reduction in cortical thickness/volume in the S1 cortex; however, the fact that NAA:Cr is unaltered may indicate that neuronal function remains preserved (7, 8).

1H-MRS studies have widely been performed in chronic pain states of various aetiologies, painful-DPN remains understudied. In general, a reduction in NAA is demonstrated in different brain regions, including the thalamus, in chronic pain disorders, such as painful-DPN (11), neuropathic pain of various aetiologies (31), trigeminal neuralgia (32) and neuropathic pain due to spinal cord injury (33). However, not all studies have demonstrated a reduction in NAA in chronic pain conditions in the thalamus, for example a recent systematic review found NAA reduced in a number of brain regions in chronic back pain, but not within the thalamus (34). It is unknown as to whether there are different cerebral alterations among different neuropathic pain conditions. The findings of this study may reflect unique cerebral changes relating to painless- and painful-DPN. We believe the findings of our results are robust for a number of reasons. Firstly, the study numbers are very large for a neuroimaging study. Additionally, painless- and painful-DPN groups were well matched, with detailed clinical and neurophysiological assessments performed, and the 1H-MRS data analysis performed in a blinded manner.

The NAA:Cr reduction in painless- but not painful DPN gives new insight into the cerebral mechanisms of neuropathic pain in diabetes. These findings of this study are consistent with previous findings in pre-clinical models suggesting a relative preservation in neuronal activity at the thalamus in painful- compared to painless-DPN (35, 36). Additionally, we previously described thalamic hyperperfusion in painful- compared with painless-DPN (6), potentially as a response to elevated oxygen demand due to increased neuronal activity. Furthermore, a recent study identified participants with painless- to have a lower thalamic volume than those with painful-DPN (37). Overall, these findings suggest a preservation of function within the thalamus in painful-DPN. Persisting nociceptive inputs from the periphery may have a protective effect upon thalamic neurons. Indeed, thalamic function may be a prerequisite for the perception of neuropathic pain in DPN. Further multi-modal MR studies are required to determine the mechanism underlying the preservation of thalamic function in painful-DPN.

There are some limitations to this study. Firstly, the study only recruited males, which limits the generalizability of the results. Males only were recruited to standardize the cohort due to sex differences in cranial size. This limitation should be addressed in future studies, as there is evidence of sex differences in painful neuropathies, which has been ill explored in DPN. Also, there is a group difference in patients with subclinical-DPN who had a shorter duration of DM, were younger and had a lower blood pressure. This could confound for the NAA:Cr ratio in the thalamus in this group; however, the painful- and painless-DPN were well matched for demographic, biochemical and neurophysiological parameters. Future studies with matching of subclinical-DPN demographic parameters could advance our knowledge of the early cerebral changes in DPN.

In conclusion, in this large cerebral MRS study in DPN, thalamic NAA:Cr was reduced in painless- but not subclinical- or painful-DPN, or in the S1 cortex in all groups. This finding is suggestive of thalamic neuronal dysfunction in painless- but not painful-DPN. Preservation of thalamic neuronal function may therefore be necessary for the perception of neuropathic pain in DPN. This study adds to the growing evidence that the thalamus is an important brain region in the cerebral mechanisms of painful-DPN and a potential therapeutic target for new treatments. Further multi-modal imaging studies are required to determine the mechanisms underlying these alterations within the thalamus in participants with painless-DPN.

## Data Availability

The raw data supporting the conclusions of this article will be made available by the authors, upon reasonable request.
